# Clinical measurement properties of malnutrition assessment tools for use with patients in hospitals: a systematic review

**DOI:** 10.1186/s12937-020-00613-0

**Published:** 2020-09-21

**Authors:** Yue Camille Xu, Joshua I. Vincent

**Affiliations:** 1 School of Rehabilitation Science, McMaster University, 1280 Main St W, Hamilton, ON L8S 4L8 Canada; 2 Clinical Dietitian at Bruyere Continuing Care, Ottawa, Canada

**Keywords:** Malnutrition assessment tool, Reliability, Validity, Responsiveness, Outcome measure, Systematic review

## Abstract

**Background:**

The use of malnutrition outcome measures (OM) by registered dietitians (RD) with inpatients in hospitals has increased promoting the achievement of nutritional care goals and supporting decision-making for the allocation of nutritional care resources in hospitals. There are 3 commonly used OMs: Subjective Global Assessment (SGA), Patient Generated-Subjective Global Assessment (PG-SGA) and Mini Nutritional Assessment (MNA). The purpose of this current study was to systematically review the evidence of the clinical measurement properties of malnutrition assessment tools for use with patients admitted in hospitals.

**Methods:**

MEDLINE, Cinahl, EMBASE, and PubMed were searched for articles published between 2000 and 2019. Research articles were selected if they established reliability, validity, and responsiveness to change properties of the SGA, PG-SGA and MNA tools, were written in English, and used any of these OMs as an outcome measure. Abstracts were not considered. The risk of bias within studies was assessed using the Quality Appraisal for Clinical Measurement Study (QA-CMS).

**Results:**

Five hundred five studies were identified, of which 34 articles were included in the final review: SGA (*n* = 8), PG-SGA (*n* = 13), and MNA (*n* = 13). Of the 34 studies, 8 had a quality score greater than 75%; 23 had a quality score of 40–75% and 3 studies had a quality score of less than 40%. PG-SGA was found to have excellentdiagnostic accuracy (ROC: 0.92–0.975; Sensitivity: 88.6–98%; Specificity: 82–100%), sufficient internal consistency (Cronbach’s alpha: 0.722–0.73), and strong test-retest reliability (r = 0.866). There was insufficient evidence to suggest adequate diagnostic accuracy and good inter-rater reliability for SGA. Only one study examined the minimum detectable change of MNA (MDC = 2.1).

**Conclusions:**

The evidence of validity for the existing malnutrition assessment tools supports the use of these tools, but more studies with sound methodological quality are needed to assess the responsiveness of these OMs to detect the change in nutritional status.

## Introduction

According to the World Health Organization [1], malnutrition can be caused by many different factors including under-nutrition, over-nutrition, inadequate nutrient intake, and an unhealthy diet, resulting in chronic illnesses such as diabetes, stroke, and hypertension. A recently published prospective cohort study conducted in 18 Canadian hospitals from July 2010 to February 2013 found that 45% of patients were malnourished at admission [2]. Malnutrition in inpatients is associated with adverse health outcomes, such as the development of infectious diseases, respiratory failure, and pressure ulcers [3, 4]. Moreover, the impact of malnutrition on health outcomes for patients with stroke can be significant and increase mortality and delay functional recovery [5, 6]. Patients that developed malnutrition during hospitalization required longer hospital stays, could not independently perform daily activities, and became high-cost care users at discharge [7, 8].

### What are malnutrition outcome measures?

The use of outcome measures in the health sector promotes the achievement of care goals, facilitates patient–healthcare professional communication, matches the delivery of health care to the patient’s needs, and supports the decision-making for the allocation of healthcare resources [9]. A well-developed outcome measure should consist of three primary psychometric properties: reliability, validity, and responsiveness to change [10, 11] (Table 1). In the context of malnutrition management, there are two broad categories of outcome measures used in tertiary care facilities: malnutrition screening and malnutrition assessment. Malnutrition screening is a quick and simple process in which the screening can be readily performed by nursing staff to identify patients at risk of malnutrition and inform practitioners if further clinical nutritional interventions are warranted [19]. The screening process at hospital admission is a crucial step to improve safe patient care; moreover, using a validated screening tool triggers appropriate referrals to dietitians that can assess and treat malnutrition in a timely manner to reduce overspending of resources from preventable misdiagnosis and poor patient outcomes [20].

**Table 1:** Definitions of and Cut-off values for rating the Clinical Measurement Properties described in this Review

Clinical measurement property	Rating method	Definition [10, 12, 13] and/or Cut-off value [10, 14, 15]
Inter-rater reliability		A measure of the consistency for the scores obtained between two rates that are measuring the same subject.
Test-retest reliability		A property of which the stability of the result using the same outcome measure with the same group of subjects in a repeated test.
Internal consistency		A reliability measure; ensuring all measurement items measure the same context.
	Kappa^a^	A preferred statistical measure for rating the inter-rate reliability; cut-off value: ≥ 0.70– acceptable [10, 16, 17].
	Cronbach’s α^b^	A statistical measure for rating the internal consistency; cut-off value: ≥ 0.70– acceptable [10, 18].
	ICC^c^	A statistical measure for rating the reliability; cut-off value: ≥ 0.70– acceptable [10].
Concurrent validity		A type of criterion validity; examining the consistency of the score obtained by the testing instrument compared with a gold standard.
Predictive validity		A type of criterion validity; measuring a correlation between the testing score and future events.
Construct validity	*r*^d^	An essential measurement validity which assesses whether a testing instrument measures what it was made to measure by comparing hypothetical and non-criterion parameters.
		A statistical measure for rating the correlation of linear relationship between two variables; cut-off values: > 0.75 - excellent; 0.50 to 0.75 - moderate; 0.25 to 0.49 - mild; < 0.25 – weak [14].
Diagnostic accuracy	ROC^e^	An ability to discriminate the population with targeted health condition.
		A statistical measure for expressing how well the measure can distinguish between two populations; cut-off values: 0.9 to 1.0 - excellent; 0.8 to 0.89 - very good; 0.7 to 0.79 - good; 0.6 to 0.69 - sufficient; 0.5 to 0.59 - insufficient; < 0.5 - no use [15].
Responsiveness to change		An ability to detect the changes in health outcomes following the intervention.
	MDC^f^	A statistical measure for rating the response to changes which is not due to the measurement error.

*Note.* ^a^Kappa = Cohen’s Kappa; ^b^Cronbach’s α = Cronbach’s alpha; ^c^ICC = Intraclass Correlation Coefficient; ^d^r = Pearson’s correlation coefficient; ^e^ROC = Receiver Operator Cure; ^f^MDC = minimal detectable change

Malnutrition assessment is different from malnutrition screening in that an in-depth and comprehensive evaluation of nutritional status is performed; therefore, professional training is required to conduct malnutrition assessment and this process is usually completed by a trained registered dietitian (RD) [21]. Moreover, in the field of nutritional practice, the assessment tool should not only be used to diagnose malnutrition at the initial visit, but the same tool should also be used by RDs to compare the effect of nutritional intervention and to measure nutritional outcomes at re-assessment. There are three well-studied malnutrition assessment tools available for this purpose: the Mini Nutritional Assessment (MNA) [22, 23], the Subjective Global Assessment (SGA) [24] and the Patient-Generated Subjective Global Assessment (PG-SGA) [25].

#### Mini nutritional assessment (MNA)

This tool was originally developed in 1990 to assess the nutritional status of elderly patients [22]. The full form of MNA consists of 18 scored questions that are divided into 4 categories: 1) anthropometric measurements; 2) global assessment; 3) dietary history and 4) metabolic stress [22]. The MNA generates a total score of 30. The total scores are interpreted as follows: 24–30 (normal nutritional status); 17–23.5 (at risk of malnutrition); less than 17 points (malnourished) [22].

#### Subjective global assessment (SGA)

This instrument was originally developed by Detsky et al. in 1987 to predict malnutrition in patients undergoing gastrointestinal surgery [24]. The SGA consists of two assessment features: 1) history and 2) physical examination relevant to malnutrition status [24]. The history part includes patterns of weight change, dietary history, gastrointestinal signs and symptoms, physical functionality and underlying inflammatory disease [24]. Additionally, three categories of physical assessment in relation to malnutrition are used in SGA: loss of subcutaneous fat, muscle wasting and the presence of fluid retention [24]. After the assessment, the patient is classified as either SGA-A (well-nourished), SGA-B (moderately malnourished), or SGA-C (severely malnourished) [24].

#### PatientGenerated-subjective global assessment (PG-SGA)

The PG-SGA was originally developed as an extension of the SGA tool to assess the nutritional status of patients with cancer [25]. The tool includes all SGA components and involves patients to self-report their nutritional histories [25]. Additionally, there are two new features in this instrument: first, for each item of the PG-SGA, a score of 0–4 is added, the more severe the symptoms in relation to malnutrition the higher the assigned value. Second, PG-SGA can generate a numerical score in addition to summarizing a global rating of A (well-nourished), B (moderately malnourished), and C (severely malnourished). A total score between 0 and 35 quantitatively informs the severity of malnutrition and types of intervention needed: 0–1 point indicates no need for any intervention; 2–3 points suggest education needs for the patients and family; 4–8 points indicate the need for a referral to a dietitian; and a score of 9 or more recommends an action of critical nutritional intervention [25].

### What are gaps identified in the current literatures?

A survey distributed to 125 stroke-specific health care institutions in Canada during 2008–2009 revealed that the majority of RDs did not use validated screening tools to assess the nutritional status of patients with stroke; the author also suggested that these results can be extrapolated to dietitian practice in other patient areas [26]. Encouraging the use of outcome measures among RDs is a necessary step that will move the profession toward an outcome-based practice. However, it is important that these existing malnutrition assessment tools are adequately examined for their validity prior to recommending their use in a hospital setting. Many recently published systematic reviews either invested their interests on the validation of the malnutrition screening tools [19], which is different from the malnutrition assessment tools, or did not include all three malnutrition assessment tools in their reviews [27, 28]. One systematic review study with meta-analysis assessed the validity of using SGA, PG-SGA, and MNA in the community [21]. This study applied some comprehensive searching and study appraisal strategies; however, the authors did not appraise the appropriateness of the statistical methodologies used to express the criterion validity, and not all psychometric properties were included for review in this article [21].

Therefore, the objectives of this paper are to systematically review the literature available on the clinical measurement properties of three malnutrition assessment tools, SGA, PG-SGA, and MNA, used with patients in hospitals and summarize the advantages and limitations of each assessment tool.

## Methodology

PRISMA [29, 30] checklist and flow diagram were used as a reporting guideline (Additional file 1).

### Literature search

Systematic searches were performed between the months of August 2019 and November 2019 in 4 databases: PubMed, CINAHL, ProQuest, and MEDLINE (via Ovid). Hand-searching of the key journals: Clinical Nutrition Journal, Nutrition Journal, Journal of Parenteral and Enteral Nutrition, and Nutrition in Clinical Practice were also completed. The reference lists of relevant articles were also searched to ensure a comprehensive search. The search terms were developed by following three steps. First, the author (YX) reviewed Medical Subject Heading (MeSH) terms associated with malnutrition, health care institution, and the nutritional assessment tools and ensured that these search terms were relevant to the context of this research study. Second, in consultation with another investigator (JV), essential terms of psychometric properties were added to maximize the specificity of the literature search. Third, in the review of other systematic reviews, additional context-specific terms in malnutrition assessment were considered [19, 21, 31]. Therefore, the key search terms applied in this paper included “SGA”, “subjective global assessment”, “PG-SGA”, “patient-generated subjective global assessment”, “MNA”, “mini nutrition assessment”, “nutritional assessment”, “nutrition outcome”, “malnutrition”, “protein-energy malnutrition”, “undernutrition”, “hospital”, “rehabilitation”, “subacute care”, “reliability, “validity”, and “responsiveness to change”. These terms were combined using the Boolean operator OR and AND in the search. We kept the clinical measurement terms broader to obtain maximal yield.

### Study selection

The study selection in this paper was performed by YX and VJ following two steps. First, the titles and abstracts of articles were screened to identify potentially eligible studies. Second, the full articles were retrieved and carefully reviewed to meet the study eligibility criteria.

Studies were included if they met the following including criteria: Study published in English after 2000 and conducted in tertiary care facilities, including inpatient, outpatient, sub-acute care, and rehabilitation; participants included in this study review were adults over 18 years old; studies should have assessed one or more clinical measurement properties of the studied outcome measures. Studies were excluded from this review if they were completed in primary care, community, long-term care, and intensive care units set-up, which require a nutritional assessment that is different from general practice within a hospital; if malnutrition screening tools were used (e.g. the short form of MNA, which is a screening tool, not a comprehensive assessment tool) [22]; if modified versions of the outcome measureswere used (e.g. 7-point SGA, Taiwanese-specific version of MNA-T1 and T2, Thai-version PG-SGA); and if they were systematic reviews.

### Data extraction

Data collection included the collection of the key characteristics of the literature, such as population, settings, and the location where each study was conducted. Moreover, other relevant variables, including psychometric properties examined, statistical methodologies used, and the main findings of the validation result for each article are summarized in the Additional file 2.

### Risk of bias in individual studies

The Quality Appraisal for Clinical Measurement Study (QA-CMS) was chosen to evaluate the internal validity of individual studies [12]. The QA-CMS consists of 12 items spread across 5 categories: study question, study design, measurement description, statistical analyses used, and study recommendation (Additional file 3). The quality score for each item is on a scale of 0–2, giving a total score out of 24, which is converted into a percentage [12]. Two reviewers (YX and JV), who were blinded to each other’s evaluation, independently performed the study appraisal in this review. An initial calibration review was completed, in which both the reviewers independently reviewed at least 2 articles. Then each item was discussed to clarify the meaning and interpretation of the items on the QA-CMS. After completion of the independent critical appraisal, both reviewers discussed each specific item on the QA-CMS for all the articles to obtain consensus (Additional file 4).

### Summary of statistical measures use

The cut-off points of each measure used for determining the adequacy of validation results in this review are summarized in Table 1.

## Results

### Search results and study characteristics

**Fig. 1:**
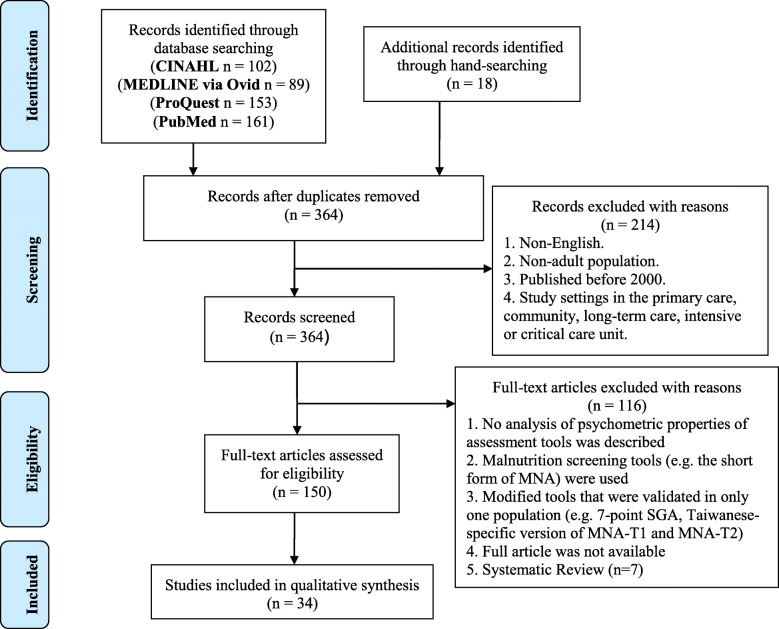
Flowchart of the systematic review study selection process

Five hundred and five studies were originally identified in the literature search: 102 in CINAHL, 89 in MEDLINE via Ovid, 153 in ProQuest, and 161 inPubMed. Additionally, 18 articles were added from the hand-searching of key journals and the reference list of identified systematic reviews. One hundred and fifty-nine articles were removed as duplicates and 214 papers were excluded after abstract screening. Following the full article review, 34 literature articles remained eligible, of which 8 investigated SGA, 13 investigated PG-SGA, and 13 investigated MNA. A flowchart of the study selection process is presented in Fig. 1.

Studies took place in Australia (*n* = 11), Turkey (*n* = 5), Japan (*n* = 3), Iran (*n* = 2), Korea (*n* = 2), Malaysia (*n* = 2), USA (*n* = 1), UK (*n* = 1), India (*n* = 1), Norway (*n* = 1), Taiwan (*n* = 1), Poland (*n* = 1), Greece (*n* = 1), Libya (*n* = 1), and Mexico (*n* = 1). The mean age of study participants was 63.26 years ranging between 19 to 92 years. The types of study designs identified in this review were prospective cohort studies (*n* = 32), a retrospective study (*n* = 1), and a randomized control trial study (*n* = 1). The psychometric properties examined in these 34 articles included test–retest reliability (*n* = 3), inter-rater reliability (*n* = 5), internal consistency (*n* = 5), predictive validity (*n* = 6), construct validity (*n* = 22), diagnostic accuracy (*n* = 7), and responsiveness to change (*n* = 3). The data extraction including pertinent study characteristics and result findings are presented in the Additional file 2.

### Risk of bias within studies

When discrepancies of scoring of specific items existed, the two reviewers (YX and JV) revisited and discussed the full article to resolve the score by mutual agreement. Of the 34 articles evaluated using QA-CMS [12], only 5 (14.70%) papers described a thorough literature review of the studied tools in their introduction, including the currently known measurement properties and the gaps identified from the current literature review that resulted in the development of relevant research questions, and calculations to determine the optimum numbers of study subjects to participate in the study were performed in 6 papers (17%). However, most papers (28 out of the 34 studies) included an adequate description of the studied instruments, scoring, and statistical methodologies applied to examine the clinical measurement properties. Thirteen articles evaluated two or more psychometric properties, of which Lin et al. [32], Ghazi et al. [33], Soysal et al. [34], and Bauer et al. [35] provided a comprehensive review of the studied tool by concurrently examining three or more indicators of reliability and validity [12]. The overall administrative procedure to perform the outcome measure in an unbiased way was evaluated primarily based on 1) if a professionally trained dietitian or experienced clinician was hired to perform patient assessments, 2) if a standardized testing procedure was followed to perform anthropometric measures, 3) if the timing when these measures were performed was consistent for each study participant, and 4) if the time-interval to assess test-retest reliability or responsiveness to change was appropriate [12].

All final scores for each eligible study are summarized in the Additional file 4. Moreover, a cut-off value of 60% was arbitrarily determined by the two authors to identify papers with an acceptable level of quality that examined the psychometric properties of the studied tools in this review.

### Synthesis of measurement properties for MNA, SGA, and PG-SGA

A summary of clinical measurement properties extracted from 18 eligible articles that obtained scores of 60% and higher based on QA-CMS are presented in Table 2.

**Table 2:** Summary of clinical measurement properties of MNA, SGA and PG-SGA tools

Measurement property	Patient population applied and measure extracted		
	MNA	SGA	PG-SGA
Diagnostic accuracy	**Geriatric patients** [36]:	**End-stage renal failure** [37]:	**Post-appendectomy surgery** [38]:
	− ROC = 0.85 ± 0.05 (95% CI 0.75 to 0.96, *p* < 0.0001); sensitivity: 83.30%; specificity: 74.40%	− SGA-C can discriminate patients who are not malnourished (specificity: 0.93-0.94), but cannot accurately identify patients with severe malnutrition (sensitivity: 0.05-0.14)	− Cut-off score = 7
			− ROC = 0.98; sensitivity: 88.60%; specificity: 100%
	**Parkinson’s disease** [33]:		**Gynecological cancer** [39]:
	− Cut-off score = 22.5	− SGA-B is not accurate enough to identify mild-moderate malnutrition (specificity: 0.61–0.65; sensitivity: 0.68–0.59)	− ROC = 0.92 (95% CI, 0.84 to 1.01, *p* < 0.001)
	− ROC =0.71 (*p* < 0.001); sensitivity: 58%; specificity: 82%		
	**Pre-frail elderly** [34]:		**Cancer inpatient** [35]:
	− Cut-off score = 25.5		− Cut-off score = 9
	− ROC = 0.83 (95% CI 0.79 to 0.87); sensitivity: 72.10%; specificity: 91.20%		− sensitivity: 98%; specificity: 82%
	**Frail elderly** [34]:		
	− Cut-off score = 25.5		
	− ROC = 0.90 (95% CI 0.88 to 0.93); sensitivity: 72.10%; specificity: 91.20%		
Test-retest reliability	**Stroke** [32]:	None	**Palliation** [40]:
	− ICC = 0.91 (95% C, 0.84 to 8.94)		− *r* = 0.87
Internal consistency	**Parkinson’s disease** [33]:	None	**Hemodialysis** [41]
	− Cronbach’s α = 0.70 (95% CI, 0.62 to 0.77)		− Cronbach’s α = 0.73
	**Geriatric patients** [34]:		**Palliation** [40]:
	− Cronbach’s α = 0.70		− Cronbach’s α = 0.72
Inter-rater reliability	None	**General medicine** [42]:	None
		Patients rated by two dietitians (Cohen’s kappa = 0.96, p < 0.001) versus dietitian and a trained allied health assistance (Cohen kappa = 0.84, p < 0.001)	
		**Gynecological cancer** [43]:	
		Patients rated by dietitian and nurse (Cohen’s kappa = 0.82)	
		**End-stage renal failure** [37]:	
		Patient rated by two dietitians (Cohen’s kappa = 0.79, 95% CI 0.67 to 0.92) versus dietitian and nephrologist (Cohen kappa = 0.60)	
Construct validity	**Dialysis** [44]:	**Digestive disease** [45]:	**Patient with cancer had undergone radiation treatment** [46]:
	Moderately correlated with a female’s body weight (*r* = 0.64) and BMI (*r* = 0.62)	Weak correlation with BMI (*r* = − 0.36), percentage of arm muscle circumference (*r* = − 0.33), percentage of triceps skin fold thickness (*r* = − 0.26), albumin (*r* = − 0.41), total cholesterol (*r* = − 0.16), and lymphocyte count (*r* = − 0.24).	
			Moderately correlated with percentage weight loss (*r* = 0.53), global quality of life at the baseline (*r* = − 0.66), and after 4 weeks of radiotherapy (*r* = − 0.61)
	**Live failure** [47]:		
	Moderately correlated with hand grip strength (*r* = 0.63) and albumin (*r* = 0.55)		
			**Cancer inpatient** [35]:
	**Cancer** [48]:		Mild correlation with weight loss in the past 6 months (*r* = 0.56)
	Highly correlated with PG-SGA at the baseline (*r* = − 0.76), at the 4–6 weeks follow up (*r* = − 0.73) and at the 8–12 weeks follow up (*r* = − 0.83).		
			**Hemodialysis** [41]:
			Moderately correlated with percentage weight loss in the past 6 months (*r* = 0.56)
	**Parkinson’s disease** [33]:		
	Mildly correlated with weight (*r* = 0.43), BMI (*r* = 0.35), mid-arm circumference (*r* = 0.268), calf circumference (*r* = 0.29)		
	**Stroke** [32]:Mildly correlated with quality of life (*r* = 0.32)		
Predictive validity	**Dialysis** [44]:	**Digestive disease** [45]:	**Cancer inpatient** [35]:
	Moderately correlated with the duration of dialysis (*r* = − 0.53)	Weak correlation with the length of hospital stays (*r* = 0.29)	Weak correlation with the length of stays (*r* = 0.36)
			**Post-appendectomy surgery** [38]:
			Weak predictive validity for the length of stays (*r* = 0.38)
Responsiveness to change and minimal detectable change (MDC)	**Stroke** [32]:	None	**Patient with cancer had undergone radiotherapy** [46]:
	MDC = 2.1		a moderate correlation between the change in PG-SGA score and change in global quality of life after 4 weeks of radiotherapy (*r* = − 0.55)

## Discussion

A comprehensive systematic review was performed to validate the clinical measurement properties of three malnutrition assessment tools: SGA, PG-SGA, and MNA. A broad range of clinical measurement properties was studied in this systematic review, including internal consistency, inter-rater reliability, test-retest reliability, construct validity, criterion validity, and responsiveness to change. Furthermore, studies conducted in diverse ethnicity and patient population groups were identified.

Approximately 30 years ago, the MNA tool was developed in recognition of the high prevalence and specificity of malnutrition among institutionalized geriatric patients [22, 23]. Nowadays, MNA is well known by health care providers for evaluating the adequacy of nutritional status in elderly patients [49], and its reliability is also confirmed in this review by an acceptable internal consistency (Cronbach’s alpha coefficient: 0.70 to 0.70) [33, 34] and test-retest reliability (ICC = 0.91, 95% CI, 0.85 to 8.94) [32]. Moreover, MNA was strongly correlated with PG-SGA for the assessment of hospitalized patients affected by stroke in Australia [48], and MNA correlated with objective measures including anthropometric and laboratory tests for the assessment of malnutrition among patients with end-stage renal failure, liver disease, gerontological conditions, and stroke in European and Asian populations [44, 47, 50–52]. Moreover, a lower MNA score predicted a longer duration of dialysis for patients with end-stage renal disease [44]. The most interesting finding about the the MNA tool was its responsiveness to change (MDC = 2.1) which indicates a higher accuracy to assist with clinician’s clinical judgment amid nutritional re-evaluation of patient outcome [32]. Finally, as a diagnostic tool, MNA was also found to have a good to excellent diagnostic accuracy (ROC:0.71 to 0.90; Sensitivity: 58 to 83.30%; Specificity: 74.40 to 91.20%) in Asian, Australian, and European patient populations [33, 34, 36].

There are many advantages to using MNA in clinical practice. For example, the tool is easy to use and a full assessment can be completed within 10 min [53]; moreover, the tool is accessible online at no cost. The study by Guigoz et al. [54] also indicatedacceptable results of this MNA tool for use among 30,000 elderly patients in various health care facilities and different countries. Furthermore, this review revealed that many recent studies have expanded their interests to validate the use of the MNA tool for patients other than geriatrics; this has expanded its popularity for use in patients with complex medical needs. However, the challenge exists in the applicability of Body Mass Index (BMI) measurements for patients in tertiary-care settings [53]. For example, there are disagreements in BMI thresholds based on different age-group; body weight is sensitive to the change in fluid status, which is commonly seen in inpatients; cancer tumors can also significantly increase the body mass, thus creating bias of BMI interpretation [55]. On the other hand, MNA contains questions to self-evaluate nutritional and health status, and this may reduce its applicability in patients with declined cognition or impaired speech capacity [53]. Finally, questions that address food choices, portion size, and the mode of feeding may not be appropriate for patients who are nutritionally stable but are receiving enteral feeding as an alternate route of food intake [56].

Among the three malnutrition assessment tools studied in this review, SGA was the first tool developed and validated for use in healthcare. Moreover, it was recommended as an acceptable assessment tool by the European Society for Clinical Nutrition and Metabolism (ESPEN) [57]. Compared with MNA and PG-SGA, the portion of patient-reported items is lower in SGA. However, multifaceted anthropometric measures are added into the assessment to examine the muscle mass loss, subcutaneous fat loss, and signs of fluid overload in 11 areas of body parts; these features of SGA were reported to improve the accuracy of malnutrition assessment [23, 58], and the tool has been validated for use in a variety of patient populations [59]. However, in the current review, SGA was not found to have sufficient sensitivity for the identification of severe malnutrition among patients with renal failure; one explanation about this conflicting finding could be that fluid overload as a result of end-stage renal disease may mask the sign of subcutaneous fat loss, which can interfere with clinicians’ subjective judgments on nutritional status [37].

One commonly reported limitation for the clinical application of SGA is its accuracy in relation to the rater’s experiences [60, 61]. Steenson et al. [62] found that dietitians with more than 5 years of clinical experience generated the highest inter-rater reliability with the benchmark compared with other groups of dietitians who had fewer years of working experiences after graduation. Therefore, inter-rater reliability is a very pertinent psychometric property to validate SGA in clinical measurement studies. Interestingly, in this review, the agreement between trained dietitians was acceptable (Cohen kappa = 0.96, *p* < 0.001) [63]; however, an inadequate agreement was found between a renal dietitian and a nephrologist (Cohen kappa = 0.60) [37]. On the other hand, the studied tool was found to correlate with anthropometric measures and biochemical measures in a variety of patient populations in Asia and Europe [45, 64]. Moreover, SGA with category B or C predicts longer hospital stays [45] and mortality [65].

The advantage of SGA is its clinical utility. It is simple, quick, inexpensive, and has been widely accepted as a criterion to validate new tools developed for nutritional screening and assessment [19]. Most interestingly, SGA was recognized as a nutritional screening tool used to increase the Diagnosis Related Group based health care reimbursement in Europe [66] and the coding of malnutrition on a casemix-based hospital funding system in Singapore [67]. However, in addition to the need for ongoing training and practice to maintain the high accuracy of malnutrition diagnosis by SGA [62], this subjective assessment tool lacks responsiveness to change to detect the changes in nutritional status following intervention [60]. This disadvantage may limit its use in clinical practice to measure the effect of malnutrition treatment and it may reduce its selection for use as an outcome measure in future nutritional studies.

The features of PG-SGA included a shift from clinician-centered to patient-centered assessment approach and enhancing patient-clinician interaction, thus promoting collaborative decision making and streamlining care delivery [68]. Moreover, the Oncology Nutrition Dietetic Practice Group of the American Dietetic Association has recognized the PG-SGA as the recommended malnutrition assessment tool for use in a patient with cancer [69]. PG-SGA was found to have outstanding diagnostic accuracy (ROC: 0.92 to 0.98; Sensitivity: 88.60 to 98%; Specificity: 82 to 100%) for the identification of malnutrition in patients with cancer or those undergoing surgery upon admission to the hospital [35, 38, 39]. Moreover, PG-SGA is a reliable tool as evidenced by several findings that have indicated acceptable internal consistency (Cronbach’s alpha = 0.72 to 0.73) in a variety of patient populations and acceptabletest-retest reliability (*r* = 0.87) witha 14-day-period reassessment [40, 41]. PG-SGA correlates with various nutritional parameters and nutritionally associated outcomes, such as global quality of life, to mostly assess patients with cancer in Australian and Asian populations [35, 41, 46, 70]. Furthermore, a higher PG-SGA score can predict longer hospital stays for patients with stroke [71], cancer [35], and undergoing gastrointestinal surgery [38]. Isenring et al. [46] reported that a change in PG-SGA score of 9 (95% CI, 7.20 to 10.90) was required to achieve improvement or deterioration of nutritional status; however, the authors did not explain if this score was validated or a standard error of measurement (SEM) was calculated. Therefore, the sensitivity to change of PG-SGA is still unclear based on the result of this review.

In addition to the patient-involved assessment feature of the PG-SGA tool, other advantages, such as the extensive consideration of nutritionally relevant disease diagnosis, the clarity of metabolic stress contributors and physical examinations, and the scoring system of this instrument have made it stand out from the other tools. Moreover, the numerical PG-SGA points feature allows the detection of changes over time; therefore, it seems to be a favorable tool used in recent nutritional studies that assessed improvement of nutritional status following the studied interventions [68, 72]. Furthermore, PG-SGA was used by Kellett et al. to identify appropriate coding of malnutrition, which allowed the estimation of unclaimed financial reimbursement based on the Diagnosis Relate Group (DRG) hospital funding criteria in Australia [73, 74]. Marshall’s study [36] also discovered a substantial agreement between the PG-SGA and the International Statistical Classification of Diseases and Health-Related Problems, 10th revision, Australian Modification (ICD-10-AM) criteria based on which Australian hospitals receive their funding reimbursements [75]; however, the method of Cohen’s kappa was misused in this study as it measures the agreement between raters, not the measures [10]. Unlike the MNA, which is a one-page streamlined assessment, the design of the PG-SGA tool is segmented and tedious. Therefore, the limitations of the use of PG-SGA in clinical practice may be that it is time-consuming to complete one assessment and labor-intensive to calculate the rating scores. Similar to the SGA tool, this scored PG-SGA also contains extensively subjective measures of physical examination on 13 areas of body parts; therefore, ongoing training and practice are also required for raters to maintain the high accuracy of malnutrition assessment and diagnosis.

### Limitations and recommendations for future studies

Although this is a comprehensive review in which we systematically appraised the quality of psychometric properties of three malnutrition assessment tools in a diverse patient population, one pertinent limitation could be the narrow timeline in the exclusion criteria. For example, Persson’s finding of the inter-rater reliability of PG-SGA was not included in this review because this study was conducted prior to 2000 [76]. Many studies that were identified in this review used a combined criterion to validate the testing tools, and these nutritional parameters are anthropometric assessments, dietary intake assessments, and biochemical measurements. However, challenges exist in nutritional studies, including recall bias, variation in appetite and nutritional symptoms, and confounding factors such as fluid overload and inflammatory effects on nutrition-sensitive markers [5, 6]. Future studies are required to include a critical appraisal tool that addresses nutrition-related confounders, bias, and sources of errors.

Finally, RDs use malnutrition assessment tools for both diagnostic and outcome measure purposes; however, only one article identified in this review appropriately applied statistical methodology to identify the responsiveness to change of the testing tool. For further studies, investigation of outcome measurements related properties, such as minimal detectable change (MDC) and minimal clinically important difference (MCID) are particularly important for both clinicians and researchers to study the treatment effect of nutritional intervention.

## Conclusions

A critical review of the clinical measurement properties of three malnutrition assessment tools for use with patients in hospitals was performed. A total of 34 studies were eligible for review, of which 18 were rated to have an acceptable quality of clinical measurement study design. The reliability and validity of all three tools: SGA, MNA, and PG-SGA were assessed; all of them were easy to use, non-invasive, and cost-effective for assessing the malnutrition status of patients.

MNA was the most validated for a variety of measurement properties, whereas SGA was the least studied tool in the last 20 years. Both MNA and PG-SGA possess acceptable test-retest reliability and internal consistency; moreover, PG-SGA had excellent diagnostic accuracy for the identification of malnutrition in various patient populations, and one study that properly examined the responsiveness to change of the MNA tool (MDC = 2.1) was identified [32]. None of the three tools showed a consistently strong correlation with other nutritional parameters and health adverse outcomes, and the inter-rater reliability for both SGA and PG-SGA was not consistently acceptable among the studies identified in this review.

Because of the lack of a gold standard to define malnutrition, this review did not find sufficient evidence to suggest the criterion (concurrent) validity for these studied tools; however, region-specific malnutrition criteria selected to identify coding of malnutrition in inpatients which informs hospital reimbursement funding may be used as a benchmark in such circumstances to validate the tool use. A future study using sound methodological quality is needed to evaluate the responsiveness to change of these malnutrition assessment tools for the detection of a change in nutritional status.

## Supplementary information

**Additional file 1.** PRISMA checklist.

**Additional file 2.** Data extraction of the identified literatures in this review [32–48, 50–52, 62–65, 70, 71, 77–84].

**Additional file 3.** Quality Appraisal for Clinical Measurement Research Reports.

**Additional file 4.** Critical Appraisal of Eligible Studies using the Quality Appraisal for Clinical Measurement Study (QA-CMS) [32–48, 50–52, 62–65, 70, 71, 77–84].

## Data Availability

All data generated or analyzed during this studyare included in this published article and its supplementaryinformation files.
