# Supplementary material for: Clinical measurement properties of malnutrition assessment tools for use with patients in hospitals: a systematic review
Source: Nutr J. 2020 Sep 21;19:106. doi: 10.1186/s12937-020-00613-0 (PMC7507822; doi:10.1186/s12937-020-00613-0)
Supplement: Supplementary file 2 — Additional file 2. Data extraction of the identified literatures in this review [32–48, 50–52, 62–65, 70, 71, 77–84]. [file 12937_2020_613_MOESM2_ESM.docx]

**Additional file 2: Data extraction of the identified literatures in this review**

| **Tool** | **Citation** | **Study Design** | **Population,**  **Study setting,**  **Location** | **Psychometric Property** | **Statistical Methodology** | **Results** |
| --- | --- | --- | --- | --- | --- | --- |
| MNA, PG-SGA | Read et al, 2005 (48) | Prospective Cohort Study | n=157,  age range: 32-81 y,  newly diagnosed cancer seen at a teaching hospital in Australia | Construct validity | Spearman's test,  p<0.01, etc. | 1. Excellent correlation was found between MNA and PG-SGA score at the baseline (*r* = -0.76, p<0.01). 2- Moderate to excellent correlations were found at the follow up between MNA and PG-SGA for the 4-6 weeks (*r* = -0.73, p<0.001) and the 8-12 weeks   (*r* = -0.83, p<0.001). |
| MNA,  PG-SGA | Marshall et al, 2016 (36) | Prospective Cohort Study | n=57,  mean age:79.1±7.3 y, geriatric patients in Geriatric Rehabilitation in Australia | Diagnostic accuracy | ROC curve;  95% CI;  Weighted-k;  Sensitivity,  Specificity;  p<0.005 level, etc. | 1- A PG-SGA score of ≥7 had an excellent sensitivity of 90% and specificity of 96.30% to identify malnutrition as identified by ICD-10-AM  2- The PG-SGA score ≥7 has excellent discriminative property to detect malnutrition as identified by ICD-10-AM (ROC AUC = 0.91 ± 0.04, 95% CI 0.84 to 0.98, p<0.0001) 3- The MNA score ≤19 has a very good discriminative property to detect malnutrition as identified by ICD-10-AM (ROC AUC = 0.85 ± 0.05, 95% CI 0.75 to 0.96, p<0.0001; Sensitivity: 83.30%; Specificity: 74.40%). |
| MNA | Ghazi et al, 2015 (33) | Prospective Cohort Study | n=143,  mean aged 55.65 y,  elderly with Parkinson's disease (PD) from an outpatient clinic in Iran | 1- Internal consistency 2- Construct validity 3- Diagnostic accuracy | Cronbach's alpha,  Correlation r,  Specificity,  Sensitivity,  95%CI,  p<.05, etc. | 1- Acceptable internal consistency of MNA used in PD population (Cronbach's alpha=0.70, 95% CI, 0.62 to 0.77). 2- Mild correlations were found between MNA total score and individual categories of weight (*r* = 0.43, p<0.001), BMI (*r* = 0.35, p<0.001), MAC (*r* = 0.27, p=0.001), calf circumference (*r* = 0.29, p=0.001) 3- Cut-off value of 26 was shown to have good discriminative property to detect BMI < 24 for malnutrition criteria in PD group (AUC=0.71, p<0.001; Sensitivity: 58%; Specificity: 82%). |
| MNA | Kuzuya et al, 2005 (50) | Prospective Cohort Study | n=226.  mean age:  78.6±0.5 y,  admitted to geriatric outpatient (n=68), hospital (n=72), nursing home (n=53) and homecare (n=33) in Japan | 1- Construct validity 2- Diagnostic accuracy | Pearson's correlation coefficient,  Specificity,  Sensitivity,  p<0.005, etc. | 1. Moderate association was found between MNA total score and BMI (*r* =0.59, p<0.0001), MAC (*r* =0.50, p<0.0001), Albumin (*r* =0.60, p<0.0001), 2- Mild association was found between TSF (*r* =0.37, p<0.0001), CC (*r* =0.28, p<0.0001), total cholesterol (*r* =0.30, p<0.0001) 3- The cutoff point below 15.5 has the maximum sensitivity (76.20%) and specificity (93.60%) to discriminate between malnutrition and risk of malnutrition in Japanese elderly (based on hypoalbuminemia and hypocholesterolemia) |
| MNA | Soysal et al, 2019 (34) | Prospective Cohort Study | n=620,  mean age:74.2±8.2 y,  elderly from Geriatric outpatient clinic in Turkey | 1- Internal consistency 2- Test-retest reliability 3- Diagnostic accuracy | Cronbach's alpha,  Intraclass correlation coefficient,  AUC ROC,  Sensitivity,  Specificity, etc. | 1. Acceptable internal consistency   (Cronbach's alpha=0.70). 2- Inadequate test-retest reliability (ICC=0.69). 3- Cut-off point of 22.5 has an excellent discriminative property to detect malnutrition in frail elderly (ROC=0.90, 95% CI 0.88 to 0.93; Sensitivity: 72.10%; Specificity: 91.20%). 4- Cut-off point of 25.5 has a very good discriminative property to detect malnutrition in pre-frail elderly (ROC = 0.83, 95% CI 0.79 to 0.87; Sensitivity: 72.10%; Specificity: 91.20%). |
| MNA | Sarikaya et al, 2015 (51) | Prospective Cohort Study | n=236,  mean age:  76.4 ± 7.2 y,  patients admitted to geriatric medicine outpatient clinic in Turkey | Construct validity | Correlation test,  p<0.05, etc. | 1- Excellent correlation was found between MNA and MNA-short form (*r* = 0.93, p<0.001). 2- Moderate correlations were found between MNA and Hand Grip Strength (*r* = 0.58, p<0.001), Basic Activities of Daily Living (BADL) (*r* = -0.60, p<0.001), Instrumental Activities of daily Living (IADL) (*r* = 0.67, p<0.001), Mobility (*r* = 0.58, p<0.001), Mid-Upper Arm Circumference (MUAC) (*r* = 0.54, p<0.001) and calf circumference (CC) (*r* = 0.51, p<0.001). 3- Mildcorrelation were found between MNA and Mini Mental State Examination (MMSE) (*r* = 0.49, p<0.001), Geriatric Depression Scale (GDS) (*r* = -0.46, p<0.001), pre-albumin (*r* = 0.34, p<0.001), Albumin (*r* = 0.48, p<0.001). 4- Weak correlation was found between MNA and low-density lipoprotein (*r* = 0.17, p<0.022). |
| MNA | Rogowski et al, 2018 (44) | Prospective Cohort Study | n=80,  mean age:  69.68 ± 8.95 y,  patients admitted to dialysis clinic at the hospital in Poland | 1- Construct validity 2- Predictive validity | Spearman's rank correlation,  p<0.05, etc. | 1- Moderatecorrelations were found between MNAused for female and Body weight (*r* = 0.64, p<0.05) and BMI (*r* = 0.62, p<0.05).  2- Moderate correlation was found between MNA used for male and the duration of Dialysis (*r* = -0.53, p>0.05).  3- Mild correlation was found between MNA for female and the duration of Dialysis (*r* = -0.32, p<0.05). 4- Weak correlations were found between MNA for male and body weight (*r* = 0.007, p>0.05), BMI (0.18, p>0.05). |
| MNA | Yasutake et al, 2018 (47) | Prospective Cohort Study | n=77,  mean age:  68.5 ± 10.7 y,  patients with liver disease admitted to hospital in Japan | 1- Construct validity | Pearson's correlation,  p<0.05, etc. | 1- Moderate correlations were found between MNA used for patient with liver cirrhosis and the maximum grasp strength (*r* = 0.63, p<0.01) and albumin  (*r* = 0.55, p<0.01). 2- Mild correlations were found between MNA for patient with liver cirrhosis and arm circumference (*r* = 0.49, p<0.01), arm muscle circumference (*r* = 0.48, p<0.01), calf circumference (*r* = 0.37, p<0.01), CONUT score (*r* = -0.45, p<0.01), Onodera's PNI (*r* = 0.42, p<0.01), C-reactive protein (*r* = -0.30, p<0.05). 3- Weak correlations were found between MNA for patient with liver cirrhosis and body weight (*r* = 0.22, p>0.05), BMI (*r* = 0.11, p>0.05), triceps skin folds  (*r* = 0.18, p>0.05). |
| MNA | Chong et al, 2019 (52) | RCT | n=45,  mean age:  59.3 ± 10.2 y,  stroke patients admitted to hospital in Malaysia | Construct validity | Pearson test or Spearman test,  p<0.05, etc | 1- Moderate correlations were found between MNA used for patient with stroke and handgrip strength  (*r* = 0.53, p=0.000), MBI score (*r* = 0.53, p=0.000). 2- Mild correlations were found between MNA for patient with stroke and weight (*r* = 0.39, p=0.007), BMI (*r* = 0.46, p=0.002), serum Albumin (*r* = 0.44, p=0.003), MAS score (*r* = 0.48, p=0.007). 3- Weak correlations were found between MNA for patient with stroke and Total lymphocyte (*r* = 0.09, p=0.549), Dietary intake (*r* = 0.16, p=0.29), Protein intake (*r* = 0.29, p=0.055). |
| MNA | Ongun et al, 2018 (82) | Prospective Cohort Study | n=96,  mean age:  63.68 ± 6.41 y,  patients with Parkinson’s disease referred to an outpatient clinic in Turkey | Construct validity | Spearman's correlation test,  p<0.05, etc. | Moderate correlations were found between MNA score and UPDRS total score (*r* = -0.64, p<0.001), PDQ39-mobility score (*r* = -0.69, p<0.001), and HADS-depression score (*r* = -0.63, r<0.001). |
| MNA | Fereshtehnejad et al, 2014 (79) | Prospective Cohort Study | n=150,  mean age:  60.8 ± 10.8 y,  patients with Parkinson’s disease referred to outpatient clinic in Iran | Construct validity | Pearson correlation, p<0.05 | Moderate correlations were fund between MNA score and UPDRS total score (*r* = -0.61, p<0.001), PDQ39-mobility score (*r* = -0.59, p<0.001), and HADS-depression score (*r* = -0.58, r<0.001). |
| MNA | Lin et al, 2019 (32) | Prospective Cohort Study | n=59,  mean age:  71.3 ± 14.7 y,  patients with stroke admitted to a hospital in Taiwan | 1- Test-retest reliability 2- Construct validity 3- Responsiveness to change | Intraclasscorrelation coefficient,  MDC,  Pearson correlation coefficient,  95% CI,  p<0.05, etc. | 1- Excellent test-retest reliability (ICC=0.91, 95%CI, 0.85 to 8.94). 2- Minimal detectable change for MNA = 2.1. 3- Mild correlation between MNA score and the QOL (*r* = 0.32, p=0.013). |
| MNA | Murphy et al, 2000 (81) | Prospective Cohort Study | n=49,  mean aged 71.5 y ranging between  60-103 y,  patients admitted to orthopedic wards for surgery in the UK | Construct validity | Pearson's test, p<0.05, etc. | A mild correlation was found between MNA and albumin (*r* = 0.34, p=0.05). |
| SGA | Newman et al, 2018 (63) | Prospective Cohort Study | n=45,  mean aged 54.6 y, patients admitted to a rural hospital in Australia | Inter-rater reliability | Kappa,  Percent exact agreement (PEA),  p<0.001, etc. | 1- Acceptable agreement was found between RDs to rating on SGA (kappa = 0.96, PEA=92.90%, p<0.001) 2-Acceptable agreement was found between RDs and trained allied health assistance (AHA) to rating on SGA (kappa = 0.84, p<0.001, PEA=84.40%) |
| SGA | Nursal et al, 2005 (42) | Prospective Cohort Study | n=2211,  mean aged 54.3 y  patient admitted to a tertiary care hospital in Turkey | Inter-rater reliability | Kappa, p<0.001, etc. | An acceptable inter-observe agreement was found between Dietitian (>5 year of experience) versus nurse (>1 year working in nutritional support) (kappa=0.82, p<0.001) |
| SGA | Thoresen et al, 2002 (64) | Prospective Cohort Study | n=46,  mean aged 68 y,  patients with cancer in a tertiary hospital in Norway | Construct validity | Pearson's correlation coefficient,  p<0.05, etc. | 1- Excellent correlations were found between SGA categories and BMI (*r* = -0.78, p<001) and weight loss % from prediagnosis weight (*r* = 0.76, p<0.001)  2- Moderate correlations were found between SGA categories and weight (*r* = -0.68, p<0.001), muscle mass (triceps skin fold *r* = -0.64, p<0001; mid-upper arm muscle circumference r=-0.68, p<0001), and prealbumin (*r* = -0.59, p<0.001) 3- Only mild association between SGA categories and Alb (*r* = -0.45, p<0.001) |
| SGA | Cooper et al, 2002 (37) | Prospective Cohort Study | n=76,  mean age:  63.5±15.2 y,  patients with end-stage renal disease in a tertiary care hospital in Australia | 1- Inter-rater reliability 2- Diagnostic accuracy | Weighted k score,  ROC curves,  Sensitivity,  Specificity,  p<0.05, etc. | 1- Inadequate agreement was found between two observers(weighted k=0.6) who are nephrologists with 3 years of training in nutrition in patient with end-stage renal disease and a registered dietitian.  2- SGA-C has a good discriminative ability to exclude patients who don't have malnutrition (specificity, 0.93 to 0.94), but it cannot accurately classify patients into the category of severe malnutrition (sensitivity, 0.05 to 0.14).  3- SGA-B has a fair discriminative ability to identify patients who are mild to moderate malnourished (specificity, 0.61 to 0.65; sensitivity, 0.68 to 0.59). |
| SGA | Wakahara et al, 2007 (45) | Prospective Cohort Study | n=262,  mean aged 66 y,  patients with digestive diseases were admitted to a hospital in Japan | 1- Construct validity 2- Predictive validity | Spearman's correlation coefficient,  p<0.05 level, etc. | 1- Weak to mild correlations were found between SGA and other nutritional parameters: BMI, % AMC, % TSF, Albumin, Total cholesterol, Lymphocyte count (-0.36, -0.33, -0.26, -0.41, -0.16, -0.24. all is p<0.01). 2- Mild association was found between SGA and predicting hospital stays (*r* = 0.29, p<0.01) |
| SGA | Santoso et al, 2004 (43) | Prospective Cohort Study | n=67,  mean age: 51.5±12.9y,  patients with gynecological cancer admitted to hospital in the USA | Inter-rater reliability | Weighted k score,  95% CI, etc. | An acceptable agreement was found between two clinicians to rating on SGA (weighted kappa = 0.79, 95% CI 0.67 to 0.92) |
| SGA | Steenson et al, 2013 (62) | Prospective Cohort Study | n=28,  patients admitted in a public tertiary teaching hospital in Australia | Inter-rater reliability | Percentage of agreement,Cohen k statistic, etc. | Compared to exemplar Dietitian (>20 y of experience using the SGA):  1- More experienced healthcare professionals (>5 years after graduation) showed higher agreement (k=0.61-1.00) than less experienced healthcare professionals (3-5 y after graduation)(k=0.59-1.00).  2- New graduates (1-2 y) had the least agreement  (k= 0.00 - 1.00). |
| SGA | Kirushnan et al, 2017 (65) | Prospective Cohort Study | n=100,  mean age: 61±11.3 y,  patients on hemodialysis admitted to hospital in India | 1- Predictive validity 2- Construct validity | Pearson'scorrelation coefficient,  95% CI,  p<0.05, etc. | 1- Mild correlation was found between SGA-B and SGA-C and the mortality in patient receiving hemodialysis during hospitalization (*r* = 0.42, p<0.001). 2- Moderate correlation between SGA and MIS (malnutrition-inflammation score) in assessing malnutrition among patients with hemodialysis  (*r* = 0.54, p<0.001) |
| PG-SGA | Bauer et al, 2002 (35) | Prospective Cohort Study | n=71,  mean age:  57.6±15.4 y,  cancer patients admitted to oncology ward of a tertiary hospital in Australia | 1- Internal consistency 2- Predictive validity 3- Construct validity 4- Diagnostic accuracy | α-Cronbach reliability coefficient,  Sensitivity  Specificity, Correlation analysis,  p<0.05, etc. | 1- The scored PG-SGA (score of ≥9) had good sensitivity (98%) and specificity (82%) to discriminate malnutrition as diagnosed by SGA. 2- Mild correlation was found between PG-SGA and the percentage weight loss in the past 6 months  (*r* = 0.31, P=0.012) 3- Inadequate internal consistency (standardized item α-Cronbach = 0.64).  4- Mild correlation was found between the PG-SGA score and predicting length of stay (*r* = 0.3, p=0.034). |
| PG-SGA | Bauer and Capra, 2004 (78) | Prospective Cohort Study | n=8,  mean age:  55.1±5.0 y,  cancer patient admitted to a tertiary hospital in Australia | Responsiveness to change | Correlation analysis,  p<0.05 level, etc. | Excellent correlation was found between the changes in nutritional status after 8 weeks of nutritional intervention as determined by PG-SGA and the changes in quality of life (measured by the European Organisation for Research and Treatment of Cancer Quality of Life Questionnaire (EORTCQLQC30)) (*r* = -0.84, P=0.02), changes in KPS (Karnofsky performance status) (*r* = -0.88, P=0.009) and change in lean body mass (*r* = -0.99, P=0.40). |
| PG-SGA | Isenring et al, 2003 (46) | Prospective Cohort Study | n=60,  mean age:  61.9±14.0 y,  cancer patients admitted to radiation oncology center in Australia | 1-Responsiveness to change  2- Construct validity | The correlation coefficient, p<0.05 level, etc. | 1- Moderate correlation was found between the change in PG-SGA score and change in global QoL  (*r* = -0.55, P<0.001) after 4 weeks of radiotherapy. 2- Change in the PG-SGA score of +/- 9 was required (95% CI=7.2 to 10.9) to move one SGA category.  4- Moderate correlation was found between PG-SGA and the percentage weight loss (*r* = 0.53, P<0.001) 5- Moderate correlation was found between the PG-SGA score and global QoL at baseline (*r* = -0.66, P<0.001) and after 4 weeks of radiotherapy  (*r* = -0.61, P<0.001). |
| PG-SGA | Desbrow et al, 2005 (41) | Prospective Cohort Study | n=60,  mean age:  63.9±16.2 y,  patient with hemodialysis admitted to a tertiary hospital in Australia | 1- Internal consistency 2- Construct validity | α-Cronbach reliability coefficient, Sensitivity  Specificity, Spearman correlation coefficient,  p<0.05 level, etc. | 1-Moderate correlation was found between the PG-SGA score and the percentage weight loss in the past 6 months (*r* = 0.56, P<0.001). 2- Acceptable internal consistency (standardized item α-Cronbach = 0.73). |
| PG-SGA | Lim et al, 2010 (80) | Prospective Cohort Study | n=73,  mean aged 64.13 y,  patients with Cerebral Infarct admitted to HyungHee University Oriental Medical Center in Korea. | Construct validity | Pearson's correlation coefficient;  p<0.05, etc. | Moderate to Excellent correlations were found between PG-SGA and DDS (dietary diversity score), DQI-I (dietary quality Index-International), DVS (dietary variety score) at *r* = -0.65, -0.69, -0.83 (P=0.000) |
| PG-SGA | Martineau et al, 2005 (71) | Retrospective Study | n=73,  mean age:  72± 12.9 y,  inpatient stroke unit in a tertiary hospital in Australia | Predictive validity | Correlation coefficient;  p<0.05 level, etc. | A moderate correlation was found between the PG-SGA score and the length of stay (*r* = 0.54, P<.001) |
| PG-SGA | Tsilika et al, 2015 (40) | Prospective Cohort Study | n=238,  mean age:  65.9± 9.6 y  patients with cancer admitted to thepalliative care unit in Greece | 1-Internal consistency 2-Test-retest reliability | α-Cronbach reliability coefficient,  Pearson's r correlation coefficient,  p<0.0005 level, etc. | 1- Acceptable internal consistency (α-Cronbach = 0.72) 2- Acceptable test-retest reliability (*r* = 0.87, P=non-significant by paired t-test) , |
| PG-SGA | Na et al, 2018 (70) | Prospective Cohort Study | n=1,588,  mean age: 61.1±11 y,  cancer patients admitted to National Cancer Center in Korea | Construct validity | Spearman's correlation coefficent,  p<0.05 level, etc. | A moderate correlation was found between total PG-SGA and patient's food intake (*r* = 0.74, p<0.05). |
| PG-SGA | Huang et al, 2014 (38) | Prospective Cohort Study | n=86,  mean age:  41.9 ±14.8 y,  patients had undergone appendectomy surgery within 24 hrs of admission in Malaysia | 1- Predictive validity 2- Diagnostic Accuracy | Spearman rank correlation,  p<0.10, etc. | 1- Mild correlation was found between the PG-SGA score and the length of stay (*r* =0.38, p<0.001) 2- The cut-off point of 7 shows the highest accuracy to predict the global classification of well nutrition and malnutrition, with a sensitivity of 86.67% and the specificity is 100%, and excellent (ROC=0.98). |
| PG-SGA | Pandey et al, 2011 (83) | Prospective Cohort Study | n=200,  mean age:  50.8 ± 13.5 y,  patients with cancer undergoing chemotherapy and/or radiotherapy in Libya hospital | Construct validity | Pearson's correlation coefficient;  p<0.05, etc. | Moderate correlation was found between PG-SGA score and the weight (*r* = 0.64), food intake (*r* = 0.68), symptoms (*r* = 0.91), functional capability (*r* = 0.62), history (*r* = 0.96), co-morbid condition (*r* = 0.58), metabolic stress (*r* = 0.60) and physical examination (*r* = 0.51). |
| PG-SGA | Alkan et al, 2018 (77) | Prospective Cohort Study | n=104,  mean aged 53.3 y, patients with gastrointestinal system cancer admitted to hospital in Turkey | Construct validity | Spearman correlation coefficient,  p<0.05, etc. | A weak correlation was found between PG-SGA score and handgrip strength (HGS) (*r* = -0.12, p= 0.071) |
| PG-SGA | Valente et al, 2019 (84) | Prospective Cohort Study | n=80,  mean age: 60.8 ± 13.5 y,  patients with cancer admitted to hospital for an operation in Mexico | Construct validity | Pearson's correlation coefficient;  p<0.05, etc. | Weak correlation was found between PG-SGA and the adductor pollicis muscle thickness in dominant hand (DAPMT) (r= -0.26), the adductor pollicis muscle thickness in non-dominant hand (NDAPMT) (*r* = -0.25), the dominant handgrip strength (DHGS)  (*r* = -0.33), the non-dominant handgrip strength (NDHGS) (*r* = -0.31). All p<0.05 |
| PG-SGA | Laky et al, 2008 (39) | Prospective Cohort Study | n=194,  mean age: 58.7±14.4 y, gynecologic oncology patients admitted to hospital in Australia | Diagnostic Accuracy | ROC AUC,  95% CI,  p<0.05, etc. | PG-SGA was found to have anexcellent discriminative diagnosis for malnutrition as compared with SGA (ROC=0.92, 95% CI, 0.83 to 1.01, p<0.001) |
