# Supplementary material for: Clinical measurement properties of malnutrition assessment tools for use with patients in hospitals: a systematic review
Source: Nutr J. 2020 Sep 21;19:106. doi: 10.1186/s12937-020-00613-0 (PMC7507822; doi:10.1186/s12937-020-00613-0)
Supplement: Supplementary file 3 — Additional file 3. Quality Appraisal for Clinical Measurement Research Reports. [file 12937_2020_613_MOESM3_ESM.docx]

**Quality Appraisal for Clinical Measurement Research Reports**

**Evaluation Form**

Authors: _____________________________ Year: ___________________ Rater: ____

*Use this form to rate the quality of a clinical measurement study. To decide which score to provide for each item on your quality checklist, pick the descriptor that sounds most like what was reported in the study you are evaluating. Items rank descriptors are provided in the guide. (Forms and guides to extract study data for evidence synthesis are available from developer at macderj@mcmaster.ca)*

| **Evaluation criteria** | **Score** | | |
| --- | --- | --- | --- |
| Study question | 2 | 1 | 0 |
| 1. Was the relevant background work cited to define what is currently known about the measurement properties of measures under study, and the potential contributions of the current research question to informing that knowledge base? |  |  |  |
| Study Design |  |  |  |
| 2. Were appropriate inclusion/exclusion criteria defined? |  |  |  |
| 3. Were specific clinical measurement questions/hypotheses identified? |  |  |  |
| 4. Was an appropriate scope of measurement properties considered? |  |  |  |
| 5. Was an appropriate sample size used? |  |  |  |
| 6. Was appropriate retention/follow-up obtained? (for studies involving retesting; otherwise n/a) |  |  |  |
| Measurements |  |  |  |
| 7. Were specific descriptions provided of the measure under study and the method(s) used to administer it? |  |  |  |
| 8. Were standardized procedures used to administer all study measures in a manner that minimized potential sources of error/bias (including the study measure and its comparators)? |  |  |  |
| **Analyses** |  |  |  |
| 9. Were analyses conducted for each specific hypothesis or purpose? |  |  |  |
| 10. Were appropriate statistical tests performed to obtain point estimates of the measurement properties? |  |  |  |
| 11. Were appropriate ancillary analyses done to quantify the confidence in the estimates of the clinical measurement property (Precision/Confidence intervals; benchmark comparisons/ROC curves, alternate forms of analysis like SEM/MID, etc.)? |  |  |  |
| Recommendations |  |  |  |
| 12. Were clear, specific and accurate conclusions made about the clinical measurement properties; that were associated with appropriate clinical measurement recommendations and supported by the study objectives, analysis and results? |  |  |  |
| **Subtotals** (of columns 1 and 2) |  |  |  |
| **Total score** (sum of subtotals/24*100);  if for a specific paper or topic an item is deemed inappropriate then you can sum of items/2*number of items *100 |  |  |  |

*From:* MacDermid J. Quality appraisal for clinical measurement studies - evaluation form and guidelines. In: Law MC, MacDermid J, editors. Evidence-Based Rehabilitation: A Guide to Practice. 3rd ed. Thorofare, NJ: Slack; 2014.
