# Supplementary material for: Clinical measurement properties of malnutrition assessment tools for use with patients in hospitals: a systematic review
Source: Nutr J. 2020 Sep 21;19:106. doi: 10.1186/s12937-020-00613-0 (PMC7507822; doi:10.1186/s12937-020-00613-0)
Supplement: Supplementary file 4 — Additional file 4. Critical Appraisal of Eligible Studies using the Quality Appraisal for Clinical Measurement Study (QA-CMS) [32–48, 50–52, 62–65, 70, 71, 77–84]. [file 12937_2020_613_MOESM4_ESM.docx]

**Additional file 4:** Critical Appraisal of Eligible Studies using the Quality Appraisal for Clinical Measurement Study (QA-CMS)

| **Studies** | **Tools** | **Item #1** | **#2** | **#3** | **#4** | **#5** | **#6** | **#7** | **#8** | **#9** | **#10** | **#11** | **#12** | **Total Score** |
| --- | --- | --- | --- | --- | --- | --- | --- | --- | --- | --- | --- | --- | --- | --- |
| Read et al, 2005 (48) | MNA,  PG-SGA | 1 | 1 | 0 | 1 | 1 | n/a | 2 | 2 | 2 | 2 | 0 | 2 | **63.60%** |
| Marshall et al, 2016 (36) | MNA,  PG-SGA | 1 | 2 | 2 | 0 | 0 | n/a | 2 | 2 | 2 | 2 | 2 | 1 | **72.70%** |
| Ghazi et al, 2015 (33) | MNA | 2 | 2 | 1 | 2 | 1 | n/a | 2 | 2 | 2 | 2 | 2 | 2 | **90.90%** |
| Kuzuya et al, 2005 (50) | MNA | 1 | 2 | 1 | 1 | 1 | n/a | 2 | 2 | 1 | 2 | 0 | 0 | **59.09%** |
| Soysal et al, 2019 (34) | MNA | 1 | 2 | 2 | 2 | 2 | 0 | 1 | 0 | 1 | 2 | 2 | 2 | **70.80%** |
| Sarikaya et al, 2015 (51) | MNA | 1 | 1 | 1 | 0 | 1 | n/a | 2 | 2 | 1 | 2 | 0 | 1 | **54.50%** |
| Rogowski et al, 2018 (44) | MNA | 1 | 2 | 2 | 1 | 0 | n/a | 2 | 2 | 2 | 2 | 0 | 1 | **68.18%** |
| Yasutake et al, 2018 (47) | MNA | 1 | 1 | 2 | 0 | 0 | n/a | 2 | 2 | 2 | 2 | 0 | 2 | **63.60%** |
| Chong et al, 2019 (52) | MNA | 1 | 2 | 0 | 0 | 2 | n/a | 2 | 2 | 1 | 2 | 0 | 0 | **54.50%** |
| Ongun et al, 2018 (82) | MNA | 0 | 2 | 1 | 0 | 0 | n/a | 2 | 2 | 2 | 2 | 0 | 2 | **59.09%** |
| Fereshtehnejad et al, 2014 (79) | MNA | 1 | 2 | 0 | 0 | 1 | n/a | 2 | 1 | 1 | 2 | 0 | 1 | **50.00%** |
| Lin et al, 2019 (32) | MNA | 2 | 2 | 2 | 2 | 0 | 2 | 2 | 2 | 2 | 2 | 2 | 2 | **91.60%** |
| Murphy et al, 2000 (81) | MNA | 1 | 2 | 2 | 0 | 0 | n/a | 2 | 1 | 2 | 2 | 0 | 1 | **59.09%** |
| Newman et al, 2018 (63) | SGA | 1 | 2 | 2 | 0 | 0 | n/a | 2 | 2 | 2 | 2 | 1 | 2 | **72.70%** |
| Nursal et al, 2005 (42) | SGA | 1 | 2 | 1 | 0 | 2 | n/a | 2 | 2 | 2 | 2 | 0 | 0 | **63.60%** |
| Thoresen et al, 2002 (64) | SGA | 1 | 2 | 1 | 0 | 0 | n/a | 2 | 1 | 2 | 2 | 0 | 2 | **59.09%** |
| Cooper et al, 2002 (37) | SGA | 1 | 1 | 2 | 1 | 0 | n/a | 2 | 2 | 2 | 2 | 2 | 2 | **77.20%** |
| Wakahara et al, 2007 (45) | SGA | 1 | 0 | 2 | 1 | 1 | n/a | 2 | 2 | 2 | 2 | 0 | 2 | **68.20%** |
| Santoso et al, 2004 (43) | SGA | 1 | 1 | 2 | 0 | 2 | n/a | 2 | 2 | 2 | 2 | 2 | 1 | **77.20%** |
| Steenson et al, 2013 (62) | SGA | 0 | 1 | 2 | 0 | 0 | n/a | 1 | 2 | 2 | 2 | 0 | 1 | **50.00%** |
| Kirushnan et al, 2017 (65) | SGA | 1 | 2 | 0 | 1 | 1 | n/a | 1 | 2 | 2 | 2 | 0 | 1 | **59.09%** |
| Bauer et al, 2002 (35) | PG-SGA | 1 | 1 | 1 | 2 | 0 | n/a | 2 | 1 | 2 | 2 | 1 | 2 | **68.20%** |
| Bauer and Capra, 2004 (78) | PG-SGA | 0 | 2 | 0 | 0 | 0 | n/a | 1 | 1 | 2 | 2 | 0 | 0 | **36.30%** |
| Isenring et al, 2003 (46) | PG-SGA | 2 | 2 | 2 | 1 | 0 | n/a | 2 | 2 | 2 | 2 | 2 | 2 | **86.30%** |
| Desbrow et al, 2005 (41) | PG-SGA | 2 | 1 | 1 | 1 | 2 | n/a | 2 | 2 | 2 | 2 | 1 | 2 | **81.80%** |
| Lim et al, 2010 (80) | PG-SGA | 0 | 1 | 0 | 0 | 0 | n/a | 2 | 1 | 2 | 2 | 0 | 1 | **40.90%** |
| Martineau et al, 2005 (71) | PG-SGA | 2 | 1 | 2 | 0 | 0 | n/a | 2 | 1 | 2 | 2 | 0 | 1 | **59.09%** |
| Tsilika et al, 2015 (40) | PG-SGA | 1 | 2 | 1 | 1 | 1 | 0 | 2 | 2 | 2 | 2 | 2 | 2 | **75.00%** |
| Na et al, 2018 (70) | PG-SGA | 0 | 2 | 0 | 0 | 2 | n/a | 1 | 0 | 2 | 2 | 0 | 1 | **45.45%** |
| Huang et al, 2014 (38) | PG-SGA | 1 | 2 | 1 | 0 | 0 | n/a | 2 | 2 | 2 | 2 | 2 | 2 | **72.70%** |
| Pandey et al, 2011 (83) | PG-SGA | 1 | 2 | 1 | 0 | 1 | n/a | 1 | 1 | 1 | 2 | 0 | 0 | **40.90%** |
| Alkan et al, 2018 (77) | PG-SGA | 1 | 2 | 2 | 0 | 1 | n/a | 2 | 0 | 2 | 2 | 0 | 1 | **59.09%** |
| Valente et al, 2019 (84) | PG-SGA | 1 | 2 | 1 | 0 | 0 | n/a | 2 | 1 | 2 | 2 | 0 | 1 | **54.54%** |
| Laky et al, 2008 (39) | PG-SGA | 1 | 2 | 2 | 0 | 1 | n/a | 2 | 2 | 2 | 2 | 2 | 1 | **77.20%** |
